# 

**Published:** 1890-11

**Authors:** 


### ANNOUNCEMENTS.

### ILLINOIS STATE VETERINARY MEDICAL ASSOCIATION.

THIS association will hold its eighth annual meeting at the Sherman House, Chicago, on November 5th and 6th. In addition to the election of officers and other business, an attractive programme of papers on "Scrofulous Ostitis in Foals," "Castration of the Horse," "Infectious Abortion of Mares," "Navicular Disease and Neurotomy," "Parturient Eclampsia in the Mare," "Simple Fever Followed by Purura."

This meeting takes place at the same time as the "American Horse Show," which offers an additional veterinary interest in Chicago.

### IOWA STATE VETERINARY MEDICAL ASSOCIATION.

THE Iowa State Veterinary Medical Association will hold its meeting at Des Moines, Iowa, on November 13th and 14th. A large attendance is expected.

### NATIONAL HORSE SHOW ASSOCIATION OF AMERICA (Lim.).

THE annual exhibition takes place at Madison Square Garden, New York, on November 10th to 15th. As applications for entries far exceed the accommodations, a complete display of all breeds of horses will be made, which will form a valuable study for veterinarians and horsemen.

### BOOKS AND PAMPHLETS RECEIVED.

The *Australian Veterinary and Live Stock Journal.* A quarterly journal devoted to the advancement of veterinary science, and to the interests of live stock in the Australian colonies. Edited by W. T. Kendall, M.R.C.V.S., Principal of the Melbourne Veterinary College. Subscription 10s. per annum, post free.

"On A New Genus of Colubridœ from Florida." By Arthur Erwin Brown. Reprinted from the Proceedings of the Academy of Natural Sciences, Philadelphia.

*North American Fauna*, Nos. 3 and 4. Edited by C. Hart Merriam, M.D,, U. S. Department of Agriculture, 1890.

Transactions of the American Dermatological Association thirteenth annual meeting, held at Boston.

Eighteenth Annual Announcement and Catalogue, Boston University School of Medicine.

Bulletin de L' Academie Royale de Medecine de Belgique, Bruxelles.

Annales et Bulletin de la Société de Medecine de Gand.

